# Searching for the genes driving assortative mating

**DOI:** 10.1371/journal.pbio.3000108

**Published:** 2019-02-07

**Authors:** Erica L. Westerman

**Affiliations:** Department of Biological Sciences, University of Arkansas, Fayetteville, Arkansas, United States of America

## Abstract

Animals display an astonishing array of diverse colors and patterns, and animals also exhibit preferences for these diverse, species-specific traits when choosing a mate (i.e., assortative mate preference). It is hypothesized that in order for both preference and trait to be species specific, alleles for a trait and the preference for that trait must be inherited together and hence maintained as linked loci. This linkage could be maintained by three different genetic architectures: (A) the genes responsible for a species-specific preferred trait also directly influence preference for that trait; (B) genes producing preference and the preferred trait are not identical but are instead in close physical proximity in the genome; and (C) genes for preference and the preferred trait are nonadjacent but are inherited together due to selection. Merrill and colleagues test these hypotheses by performing large-scale genetic mapping of mating behavior using hybrids of two sympatric species of *Heliconius* butterflies, *Heliconius melpomene* and *H*. *cydno*. They identified three small genomic regions highly associated with mate preference, one of which was adjacent to a gene for the preferred trait, and two of which were not. Their findings illustrate that mate preference may be influenced by a small number of genes, while providing support for multiple hypotheses for the genetic architecture of assortative mate preferences.

## Abstract 2

This Primer explores how a study of courting behavior in Heliconius butterflies sheds light on the genetic architecture of reproductive isolation, and provides new insights into the often elusive genes for mate preference.

Humans have been interested in describing and defining species since the days of Aristotle. For the last millennia, two questions related to species delimitation and speciation that have plagued scientists are which animals choose to interbreed, and why animals that could interbreed choose not to do so. One behavior often associated with the maintenance of species boundaries is assortative mate preference, in which individuals preferentially mate with individuals of the opposite sex that share a number of common characteristics [1–3]. These common characteristics can range from color and morphological appearance to odor or sound [4]. Assortative mate preferences often lead to assortative mating, which is hypothesized to be a key component of speciation, because it facilitates reproductive isolation (reviewed in [5]). However, preference-driven assortative mating can only facilitate reproductive isolation if both the propensity to choose a specific mate and the traits of the chosen used by the chooser to select a specific mate are heritable. In addition, alleles driving assortative preferences and corresponding traits must be inherited together (maintained in high linkage disequilibrium [LD]) because independent inheritance of these alleles would cause a breakdown in assortative mating and subsequent reproductive isolation and speciation.

Because assortative mate preferences often play an important role in speciation, the traits used by individuals to select mates are often species specific and tend to coincide with traits we (humans) use to delimitate species. Such species-specific traits are generally heritable and genetically determined, which makes identifying the genes (and subsequent developmental pathways) responsible for their variation relatively straightforward. However, the traits used in mate selection are only half of the assortative mating story. Determining causal genetic variation for observed variation in mating preferences, the other half of the assortative mating tango, has proven much more difficult.

## The trouble with behavior: Genetics of complex, ephemeral traits

One challenge in identifying genes underlying variation in mate preference is that the trait is inherently transient, because it is only expressed, and can therefore only be measured, when an individual is actively seeking a mate (i.e., doing the “choosing”). Moreover, mate preference itself is wonderfully complex, with individuals considering combinations of multiple desirable traits when selecting a mate [6]. Therefore, researchers interested in identifying the genes associated with variation in preference must first determine two things: (1) when an individual will exhibit their preference phenotype and (2) what specific traits of the chosen are used by the chooser during the mate selection process. Only then can variation in preference be measured and related to genetic variation associated with this preference.

Substantial a priori behavioral research is required to reach this point of preference gene discovery; therefore, we are only now at the dawn of mate preference gene identification in a few well-studied systems, including *Heliconius* butterflies. Over the last 20 years, a handful of studies in *Drosophila* have illustrated how species-specific genetic variation can lead to species-specific variation in mate preference, providing confirmation that genetic variation may underlie observed species-specific variation in mate preferences and subsequent assortative mating (illustrated by [7–10]). These studies have also demonstrated that differences in preference can result from species-specific differences in perceptive ability, suggesting that preference genes may reside within specific developmental pathways related to sensory systems [11].

**Fig 1:**
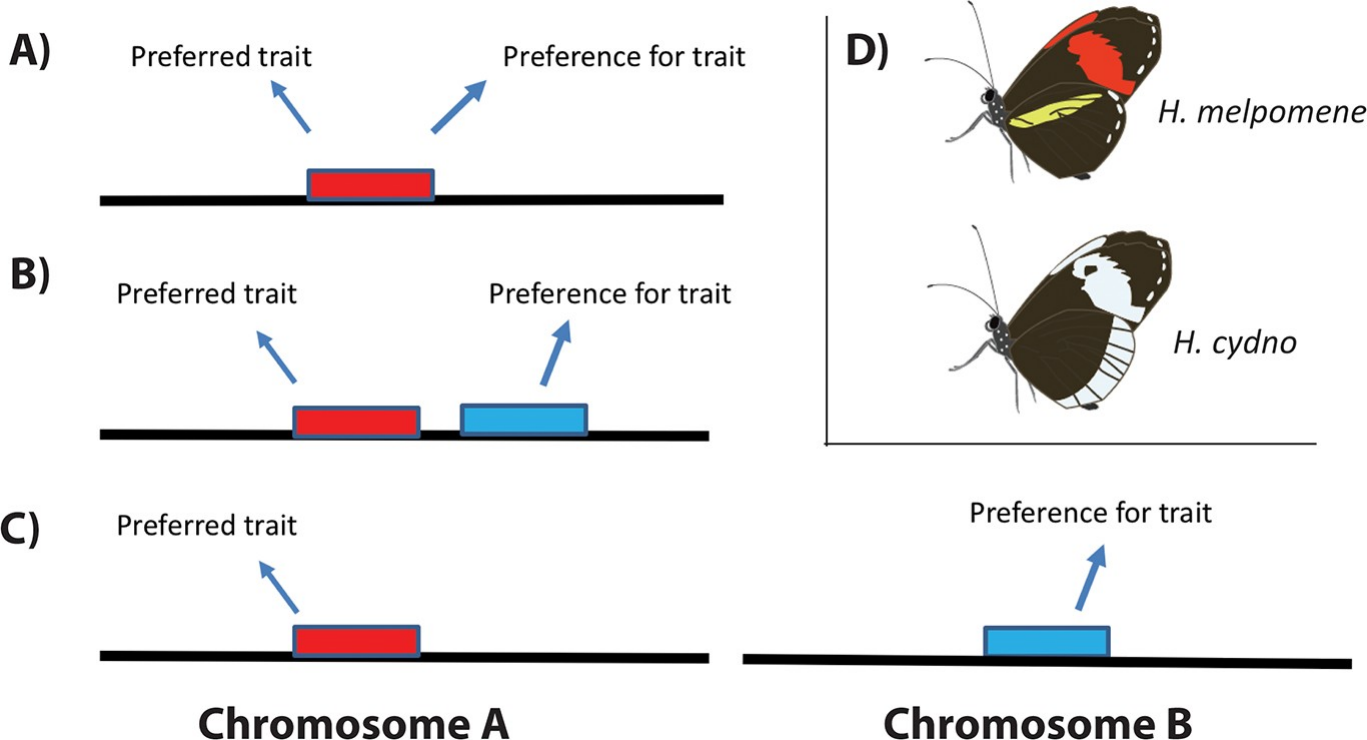
Hypothesized genetic architecture of assortative mating. (A) A single gene influences both trait and preference for that trait. (B) Two adjacent genes for trait and preference. (C) Two physically unlinked genes for trait and preference, maintained in the population due to selection. (D) Wing patterns used during mate selection in *Heliconius melpomene* and *H*. *cydno*. Butterfly drawings courtesy of M. J. Murphy.

Simultaneously, population geneticists and evolutionary biologists studying morphological variation in sister and incipient species have developed three hypotheses for the genetic architecture underlying genes for mate preference and genes for the traits animals prefer [2,3,5,12]. One hypothesis is that the same gene that controls variation in the preferred trait also has a large effect on preference for that trait (Fig 1A). Therefore, any genetic variation causing variability in the preferred trait would also lead to matching variation in preference. Alternatively, the gene influencing preference may not be the same as that influencing the preferred trait but might be located in close physical proximity, which links together alleles for the preferred trait and preference for that trait due to low genetic recombination rates (Fig 1B). A third hypothesis is that the gene for preference and the gene for the preferred trait are not physically linked but that assortative mating is so advantageous, as a result of either natural or sexual selection, that alleles for a preferred trait and preference for that trait are maintained in a population in high LD, in spite of the absence of mechanistically difficult recombination as a result of proximity (Fig 1C). (It is worth noting at this point that mate preferences in some species are learned [13]. Determining the neural and genetic mechanisms underlying learned preferences, and the effect of learned preferences on speciation, is a rich area of research beyond the scope of this primer. However, variation in learned assortative mate preferences are predicted to fit either the one- or two-gene models for genetically determined assortative mate preferences described above, depending on the mechanism underlying the learned preference [14–17], so it will not be considered as a special case here.) Determining which of the three hypothesized genetic architectures for assortative mating is most common has become one of the goals of speciation research.

## Identifying loci of large effect for assortative mate preference

Here, Merrill and colleagues [18] take advantage of recent advances in genomic sequencing and prior discoveries of the genes influencing color patterning in *Heliconius* butterflies to identify small regions of the genome highly associated with assortative mate preference (quantitative trait loci [QTLs] of large effect) in male *H*. *melpomene* and *H*. *cydno* butterflies. In these butterflies, males use the visual appearance of female wing patterns to determine which individual to approach and court. Avian predators also use the visual appearance of *Heliconius* wing patterns to assess potential butterfly toxicity and determine prey choice [19], making this a particularly interesting system for the study of genes associated with wing pattern preference and speciation because changes in wing pattern have the potential to influence both attractiveness and predation susceptibility. Male mating preferences are species specific, with *H*. *melpomene* males preferring females with red bands on their forewings, and *H*. *cydno* males preferring females with white bands on their forewings [20] (Fig 1D). The genes that determine the color of these forewing bands are known, with *optix* being largely responsible for the presence or absence of red on the forewings of *H*. *melpomene* [21], and *aristaless 1* being largely responsible for the presence or absence of white on the forewings of *H*. *cydno* [22]. Two other genes, *WntA* and *cortex*, are also known to influence color patterning in the wings of these butterflies [23,24] and therefore also have the potential to influence both preference and preferred trait in these species.

Although it may seem unrealistic to expect a single gene to influence both preference and preferred trait, wing color patterning genes in butterflies are particularly good candidates for this role. Wing color patterning genes in butterflies influence pigment production and placement (reviewed in [25,26]). Some of these same pigments are used in the eyes of butterflies and influence what wavelengths of light the butterflies see [27]. Therefore, color patterning genes have the potential to influence both butterfly wing color (preferred trait) and butterfly vision (the sensory capacity to observe said preferred trait), meaning allelic variation at these loci could concurrently influence both wing color and color preference.

To identify loci of large effect for male assortative mate preference, Merrill and colleagues first conducted a series of mate choice trials using *H*. *melpemene* and *H*. *cydno* butterflies. They then crossbred *H*. *melpomene* and *H*. *cydno* butterflies and tested the preferences of hybrid males. Lastly, they made careful use of the offspring of backcrosses (offspring of hybrids paired with either purebred *H*. *melpomene* or *H*. *cydno*) to facilitate the identification of small genomic regions highly associated with male preference for female forewing band colors. This strategy allowed them to identify three loci of large effect for male assortative mating and represents one of the first descriptions of the genetic architecture underlying assortative mating outside of *Drosophila*. Of the three large-effect loci responsible for variation in male preference, one was adjacent to, and potentially contains, the wing patterning gene *optix*, whereas the other two loci are not adjacent to any known wing patterning genes [18].

These findings are significant for a few reasons. First, they illustrate that assortative mate preference can be driven by a small number of genes of large effect. Second, they provide support for multiple hypotheses for the genetics of assortative mate preference. Future functional work is needed to determine whether *optix* influences both wing pattern and preference or whether the gene associated with that particular preference locus is adjacent to *optix*, thereby determining whether the same gene is influencing both preference and the preferred trait in this system. However, the authors have made great strides in identifying the genetic architecture underlying assortative mate preference by definitively demonstrating that genes of large effect for mate preference can be maintained in high LD with genes for the preferred trait (wing pattern in this case) in the absence of physical proximity.

## From candidate genes to neural mechanisms

Merrill and colleagues’ findings represent a large step forward in our understanding of the genetics of assortative mate preference but also highlight some remaining questions on this topic. First, in spite of concerted efforts by the research community studying assortative mate preference in a wide range of animal species—from guppies to zebra finches to *Heliconius* butterflies (see [28,29] for examples)—causative genes for preference remain elusive outside of *Drosophila*. The advent of CRISPR/Cas-9 gene editing (a relatively straightforward and inexpensive form of genetic manipulation that uses clustered regulatory interspaced short palindromic repeats (CRISPR) and the enzyme CRISPR associated 9 (Cas-9), components of the bacterial immune system, to target and edit precise locations in the genome [30]) and optogenetic technology (which uses wavelengths of light outside of the visual spectrum to manipulate neural activity [31]), coupled with this and other fine mapping studies to identify candidate genes for preference, will facilitate the identification and confirmation of multiple genes for mate preference in the coming years. Second, the identification of candidate genes for mate preference offers new insights into the types of developmental change necessary for a change in preference. It is currently unclear whether shifts in preference are the result of changes in perceptive ability, such as differences in the relative number of photoreceptors or chemoreceptors in the peripheral nervous system or changes in valence (i.e., positive or negative association) attributed to the formerly preferred trait, which may manifest as changes in synaptic strength or architecture in higher processing areas of the brain. Pheromone preferences of different species of *Drosophila* provide evidence for both [8], but there are scant data in support of either hypothesis in other animal systems. This area of neurobiology and speciation research is rich with promise, and the identification of candidate loci via fine-scale associative mapping studies such as Merrill and colleagues will inform hypotheses in this field for years to come.

In addition to the outstanding questions on the genes and neurological mechanisms underlying assortative mating and preference diversity described above, the fundamental question of the primary genetic architecture underlying assortative mate preference and speciation events remains: which genetic architecture is most commonly associated with assortative mate preference and subsequent speciation events? Is it (A) single gene for trait and preference, (B) two adjacent genes for trait and preference, or (C) two physically unlinked genes maintained in high LD? This is perhaps the low-hanging fruit of big questions in assortative mate preference research and will hopefully be answered in the near future as genomic data become available for numerous sister species with previously described assortative mate preferences.

## Abbreviations

CRISPR/Cas-9: clustered regulatory interspaced short palindromic repeats/CRISPR-associated 9
LD: linkage disequilibrium
QTL: quantitative trait locus

## References

[1] Lewontin R, Kirk D, Crow J. Selective mating, assortative mating, and inbreeding: definitions and implications Eugenics Quarterly. 1968;15(2):141–3. 10.1080/19485565.1968.9987764 5702329

[2] Felsenstein J. Skepticism towards Santa Rosalia, or why are there so few kinds of animals? Evolution. 1981;35:124–38. 10.1111/j.1558-5646.1981.tb04864.x 28563447

[3] Smadja CM, Butlin RK. A framework for comparing processes of speciation in the presence of gene flow Molecular Ecology 2011;20:5123–40. 10.1111/j.1365-294X.2011.05350.x 22066935

[4] Hebets EA, Papaj DR. Complex signal function: developing a framework of testable hypotheses. Behavioral Ecology and Sociobiology. 2005;57:197–214.

[5] Servedio MR. The role of linkage disequilibrium in the evolution of premating isolation Heredity. 2009;102:51–6. 10.1038/hdy.2008.98 18813328

[6] Andersson M. Sexual Selection. Princeton, NJ: Princeton University Press; 1994. 559 p.

[7] Dickson BJ. Wired for sex: The neurobiology of *Drosophila* mating decisions. Science. 2008;322:904–9. 10.1126/science.1159276 18988843

[8] Fan P, Manoli DS, Ahmend OM, Chen Y, Aragwal N, Kwong S, et al Genetic and neural mechanisms that inhibit *Drosophila* from mating with other species. Cell. 2013;154:89–102. 10.1016/j.cell.2013.06.008 23810192 PMC3823234

[9] Gompel N, Prud'homme B. Chemical love. Nature. 2009;461:887–8. 10.1038/461887a 19829359

[10] Etges WJ, Cardoso de Oliveira C, Gragg E, Ortiz-Barrientos D, Noor MAF, Ritchie MG. Genetics of incipient speciation in *Drosophila mojavensis*. I. Male courtship song, mating success, and genotype x environment interactions. Evolution. 2007;61(5):1106–19. 10.1111/j.1558-5646.2007.00104.x 17492965

[11] Datta SR, Vasconcelos ML, Ruta V, Luo S, Wong A, Demir E, et al The *Drosophila* pheromone cVA activates a sexually dimorphic neural circuit. Nature. 2008;452 10.1038/nature06808 18305480

[12] Verzijden MN, Lachlan RF, Servedio MR. Female mate-choice behavior and sympatric speciation. Evolution. 2005;59(10):2097–108. 16405155

[13] Verzijden MN, ten Cate C, Servedio MR, Kozak GM, Boughman JW, Svensson EI. The impact of learning on sexual selection. Trends in Ecology and Evolution. 2012;27(9):511–9. 10.1016/j.tree.2012.05.007 22705159

[14] Tramm NA, Servedio MR. Evolution of mate-choice imprinting: Competing strategies. Evolution. 2008;62(8):1991–2003. 10.1111/j.1558-5646.2008.00419.x 18485111

[15] Irwin DE, Price T. Sexual imprinting, learning and speciation. Heredity. 1999;82:347–54. 10383652 10.1038/sj.hdy.6885270

[16] Laland KN. On the evolutionary consequences of sexual imprinting. Evolution. 1994;48(2):477–89. 10.1111/j.1558-5646.1994.tb01325.x 28568308

[17] Servedio MR, Noor MAF. The role of reinforcement in speciation: theory and data. Annual Review of Ecology, Evolution, and Systematics. 2003;34:339–64. 10.1146/annurev.ecolsys.34.011802.123412

[18] Merrill RM, Rastas P, Martin SH, Melo MC, Barker S, Davey J, et al Genetic dissection of assortative mating behavior. PLoS Biol. 2019;17(2):e2005902 10.1371/journal.pbio.2005902 PMC6366751 30730873

[19] Finkbeiner SD, Briscoe AD, Reed RD. Warning signals are seductive: Relative contributions of color and pattern to predator avoidance and mate attraction in *Heliconius* butterflies. Evolution. 2014;68(12):3410–20. 10.1111/evo.12524 25200939

[20] Jiggins CD, Naisbit RE, Coe RL, Mallet J. Reproductive isolation caused by colour pattern mimicry Nature. 2001;411:302–5. 10.1038/35077075 11357131

[21] Reed RD, Papa R, Martin A, Hines HM, Counterman BA, Pardo-Diaz C, et al *optix* drives the repeated covergent evolution of butterfly wing pattern mimcry. Science 2011;333:1137–41. 10.1126/science.1208227 21778360

[22] Westerman EL, VanKuren N, Massardo D, Buerkle N, Tenger-Trolander A, Zhang W, et al *Aristaless* controls butterfly wing color variation used in mimicry and mate choice Current Biology. 2018;28(21):3469–74.e4. 10.1016/j.cub.2018.08.051 30415702 PMC6234856

[23] Martin A, Papa R, Nadeau NJ, Hill RI, Counterman BA, Halder G, et al Diversification of complex butterfly wing patterns by repeated regulatory evolution of a *Wnt* ligand. Proceedings of the National Academy of Sciences. 2012;109(31):12632–7. 10.1073/pnas.1204800109 22802635 PMC3411988

[24] Nadeau NJ, Pardo-Diaz C, Whibley A, Supple MA, Saenko SV, Wallbank RWR, et al The gene *cortex* controls mimicry and crypsis in butterflies and moths Nature. 2016;534:106–10. 10.1038/nature17961 27251285 PMC5094491

[25] Kronforst MR, Young LG, Kapan DD, McNeely C, O'Neill RJ, Gilbert LE. Linkage of butterfly mate preference and wing color preference cue at the genomic location of *wingless*. Proceedings of the National Academy of Sciences of the United States of America. 2006;103(17):6575–80. 10.1073/pnas.0509685103 16611733 PMC1458925

[26] Kronforst MR, Papa R. The functional basis of wing patterning in *Heliconius* butterflies: The molecules behind mimicry. Genetics. 2015;200:1–19. 10.1534/genetics.114.172387 25953905 PMC4423356

[27] Stavenga DG. Reflections on colourful ommatidia of butterfly eyes. Journal of Experimental Biology. 2002;205:1077–85. 11919267 10.1242/jeb.205.8.1077

[28] Wellenreuther M, Svensson EI, Hansson B. Sexual selection and genetic colour polymorphisms in animals. Molecular Ecology. 2014;23:5398–414. 10.1111/mec.12935 25251393

[29] Woodgate JL, Buchanan KL, Bennett ATD, Catchpole CK, Birghton R, Leitner S. Environmental and genetic control of brain and song structure in the zebra finch. Evolution 2014;68(1):230–40. 10.1111/evo.12261 24102614

[30] Ran FA, Hsu PD, Wright J, Agarwala V, Scott DA, Zhang F. Genome engineering using the CRISPR-Cas9 system. Nature Protocols. 2013;8:2281–308. 10.1038/nprot.2013.143 24157548 PMC3969860

[31] Fenno L, Yizhar O, Deisseroth K. The development and appilication of optogenetics Annual Review of Neuroscience 2011;34:389–412. 10.1146/annurev-neuro-061010-113817 21692661 PMC6699620

